# Bilevel Optimization-Based Time-Optimal Path Planning for AUVs

**DOI:** 10.3390/s18124167

**Published:** 2018-11-27

**Authors:** Xuliang Yao, Feng Wang, Jingfang Wang, Xiaowei Wang

**Affiliations:** College of Automation, Harbin Engineering University, Nantong Road No. 145, Harbin 150001, China; wangfeng3561@hrbeu.edu.cn (F.W.); wjfyjs550@126.com (J.W.); wangxiaowei@hrbeu.edu.cn (X.W.)

**Keywords:** autonomous underwater vehicle (AUV), bilevel optimization (BIO), energy-optimal path, path planning

## Abstract

Using the bilevel optimization (BIO) scheme, this paper presents a time-optimal path planner for autonomous underwater vehicles (AUVs) operating in grid-based environments with ocean currents. In this scheme, the upper optimization problem is defined as finding a free-collision channel from a starting point to a destination, which consists of connected grids, and the lower optimization problem is defined as finding an energy-optimal path in the channel generated by the upper level algorithm. The proposed scheme is integrated with ant colony algorithm as the upper level and quantum-behaved particle swarm optimization as the lower level and tested to find an energy-optimal path for AUV navigating through an ocean environment in the presence of obstacles. This arrangement prevents discrete state transitions that constrain a vehicle’s motion to a small set of headings and improves efficiency by the usage of evolutionary algorithms. Simulation results show that the proposed BIO scheme has higher computation efficiency with a slightly lower fitness value than sliding wavefront expansion scheme, which is a grid-based path planner with continuous motion directions.

## 1. Introduction

Autonomous underwater vehicles (AUVs) are frequently employed to perform environmental monitoring and exploration tasks, such as surveillance of the dynamics of plankton assemblages, temperature, and salinity profiles, and the onset of harmful algal blooms [1,2,3]. Path planning is of primary importance to the safety and efficient operation of a vehicle in such tasks. AUVs are frequently deployed for long periods and must operate with limited energy. Thus, a path planner should be capable of determining a trajectory that safely guides an AUV from its initial or current position to its destination with minimal energy or time. By selecting an appropriate trajectory, the path planner may enable the AUV to bypass adverse currents, exploit favorable currents and subsequently achieve high speeds while substantially saving energy. Many researchers investigated the AUV path planning problem in recent years. Here, we discuss those contributions that relate directly to our work, and these are grid-based path planning in anisotropic environments. Some of these studies use heuristic search algorithms, such as A* algorithm and the extensions of A* algorithm to the AUV path planning problem. A* method is usually used for obtaining an energy-optimal path for the AUV [4,5,6] and has been proven to be efficient. The use of the EEA* algorithm [7], which is an extension of the A* algorithm, is proposed for the planning of energy efficient paths for a marine surface vehicle when heading angle is considered. However, the above-mentioned approaches are limited by small and discrete sets of possible transitions, which result in the generation of a suboptimal path; in some cases, no path is generated even when a feasible path exists mainly because of the issue of incompleteness (see Reference [8] for more details). Therefore, traversing is difficult in reality.

A group of metaheuristic evolution-based search algorithms has been applied for the path planning problem. Genetic algorithm (GA) [9] is utilized in the determination of an energy efficient path for an AUV encountering a strong ocean current field but limited by the discrete motion directions. Unlike the other approaches, energy/time-optimal trajectory using B-splines where the coordinates of the spline control points form the chromosome genes is represented in References [10,11]. The control points can be freely located anywhere in the search space. Methods using evolutionary algorithms with the division of search space have been recently proposed to improve search efficiency [12,13]. Although the spline curve-based path planner is not restricted by the direction of motion and is beneficial for tracking and controlling an AUV, it may be not the optimal path because curved paths increase energy or time consumption.

The fast marching (FM) algorithm [14] is another approach for solving the AUV energy-optimal path planning problem that uses a first order numerical approximation of the nonlinear eikonal equation. The upgraded version of the FM known as FM* [15] or heuristically guided FM is usually employed for the AUV path planning problem, but it is restricted to linear anisotropic cost. A sliding wavefront expansion (SWE) algorithm [8] is applied for generating an arbitrary precision path for the unmanned air vehicle (UAV) in the presence of strong current fields. In this algorithm, the incompleteness issue is solved by introducing the concept of a slider, which transforms the discrete motion model into a continuum one. However, it is expensive because of the larger search space requirement than the discrete motion model, even for 2D. To improve computation efficiency and obtain an accurate optimal path, we present a new scheme, bilevel optimization. The bilevel optimization is more suitable to solve the complex, large-scale problem than the classical methods and single level evolutionary algorithms. It is widely applied in the fields of economics [16,17,18], management [19,20], and engineering [21,22]. It provides a flexible and efficient means to solve the problem of path planning. In our research, the bilevel programming scheme decomposes the task of path planning into two parts: (1) The outer optimization problem or the upper level, which is defined as finding a free-collision channel from a starting point to a destination consisting of connected grids, and (2) the inner optimization problem or the lower level, which is defined as finding the energy-optimal path in the channel generated by the upper level. This scheme uses the ant colony algorithm (ACA) [23] as the upper level algorithm and relies on the quantum-behaved particle swarm optimization (QPSO) [24] at the lower level. This arrangement prevents discrete state transitions and guarantees the optimization of the resulting path. In fact, in the field of path planning for computer game and land robot, some algorithms with any-angle transitions have been presented, which includes the path planning and path optimization, such as (Lazy) Theta* [25,26]. This is similar to the upper level and the lower level optimization of proposed bilevel optimization (BIO) scheme. However, in studying objects, purposes, and means, there are essential differences. First, (Lazy) Theta* algorithm is mainly applied in computer games and robotics without considering current fields (such as land robot), but BIO is designed for AUV. Because of the slow speed of AUV, the influence of ocean currents cannot be ignored [27]. Second, the purpose of (Lazy) Theta* algorithm is to find a shortest path and not pass through obstacles. Nevertheless, the proposed BIO scheme is to optimize the energy consumption to enhance the navigating ability for AUV with limited energy. Usually, the shortest path is not equal to the energy-optimal path in an environment with ocean currents. Some researches [13,28] have already demonstrated this. Finally, both (Lazy) Theta* and BIO are to solve the disadvantages of discrete motion direction caused by grids. (Lazy) Theta* achieves any-angle path planning by checking line-of-sight, but it is still a vertex-to-vertex searching scheme. However, the BIO scheme first generates the channel by ACO and then finds the energy-optimal path by continuous optimization technique (QPSO) in this channel. The waypoints of BIO can be placed in any position on edge, that is, edge-to-edge searching scheme.

The remaining parts of this paper are organized as follows: Section 2 outlines the system structure involved in path planning, including path formation, path evaluation, and environment model. Section 3 introduces the bilevel optimization mechanism for path planning. The simulation tests and results generated are presented in Section 4. The concluding remarks are then presented in Section 5.

## 2. Problem Statement

The objective of a path planning system is to find an optimal path that leads an AUV traveling through the ocean environment to its destination. The ocean environment is modeled as a strong currents field with fixed and moving obstacles. The optimization criterion for the path planner is set as minimal energy consumption with collision avoidance.

### 2.1. Ocean Field Environment

The information of ocean currents can be obtained from remote observations, particularly through high-frequency radar surface current measurements and satellite observations, or from numerical forecast models. Some ocean current measurement and prediction systems have been found, such as the Regional Ocean Model System (ROMS) [29] with 1 km resolutions. The use of measured or predictive ocean models is primarily intended for large regions.

In practice, the time-optimal path planning problem for AUV operation is generally solved for long-term missions (large regions) with durations of several days and trajectory lengths of hundreds of kilometers. In addition, the AUV’s deployment usually is assumed on a horizontal plane, because vertical motions in ocean structure are generally negligible due to large horizontal scales comparing vertical [30]. Hence, in this research, the ocean model based on measured or predictive is used in 2D space. According to the resolution of the measures or the precision of the forecast model, the environment is divided into grids. In each mesh, the ocean current value is characterized by a constant, and the ocean current data are simulated by the superposition of randomly distribution eddy fields. The eddy fields in the global coordinate system are provided by the following expression:

(1) $$eddy\left\{p,size\right\}:\left\{ \begin{array}{l} f\left(x,y\right)={\left(x+p_{x}\right)}^{2}+{\left(y+p_{y}\right)}^{2}\\ c_{x}=size_{x}\cdot -\frac{\partial f}{\partial x}\cdot \frac{1}{f\left(x,y\right)}\\ c_{y}=size_{y}\cdot \frac{\partial f}{\partial y}\cdot \frac{1}{f\left(x,y\right)} \end{array}\right.$$

where $p_{x}$ and $p_{y}$ are the coordinates of eddy center p on *x*- and *y*-axes, respectively, and $size_{x}$ and $size_{y}$ determine the current strength in the directions of the *x*- and *y*-axes, respectively. The sign of $size_{x}$ must be similar to that of $size_{y}$. Their signs determine the rotation direction (clockwise or anticlockwise) of the eddy field. The model of the current field F is as follows:

(2) $$F=\sum_{i=1}^{n}eddy\left\{rand\left(p_{i}\right),rand\left(size_{i}\right)\right\}$$

where *rand*( ) represents a random function. The current field is the superposition of *n* eddies.

### 2.2. Obstacle Models

In this study, the uncertainty of the position is modeled with independent normal distribution $X_{o}∼N\left(\mu_{o},\sigma_{o}\right)$. An obstacle with low uncertainty indicates a high probability that the obstacle exists at the location. Conversely, high uncertainty indicates the absence of such an obstacle. Three kinds of obstacle modeling derived from [13,31] are considered for the assimilation of different possibilities of real situations:

1. Static obstacles with fixed uncertainty: a static obstacle’s position, although fixed, have a constant measurement uncertainty incorporated in its position.
2. Quasi-static obstacles: this group of obstacles is introduced with a fixed-center and an uncertain radius that varies over time.
3. Moving obstacles: similar to the quasi-static obstacles, the uncertainty of moving obstacles increases with time, whereas their centers will change accordingly with constant speed and direction.

In modeling obstacles with continuous observations, uncertainty is fixed and depends on the accuracy and repeatability of the measuring modalities. However, in the lack of continuous observations, the uncertainty of obstacles increases such that $\sigma_{o}$ grows linearly with time.

### 2.3. Optimization Criterion

An optimization planning criterion is used to define the evaluation function as described in Section 2.3.2. Based on this evaluation function, a fitness value is measured for each candidate path.

#### 2.3.1. Path Formation

**Figure 1:**
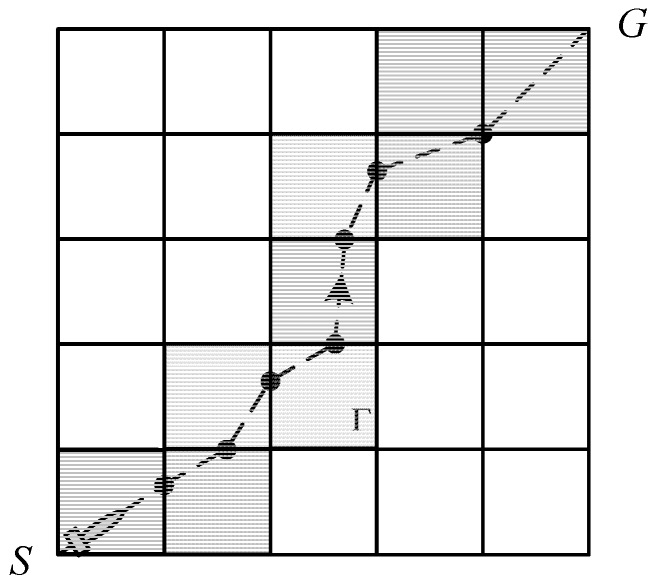
Example of a path, in which the dotted line represents path $\Gamma_{s,g}$.

In this study, a path denoted as $\Gamma_{s,g}$ is a sequence of waypoints between the initial position and a destination, in which each waypoint is located at the edge of the meshes. The waypoints are denoted as $x_{i}\;\left(i=1,2,\cdots n\right)$, then the path from *s* to *g* shown in Figure 1 is

(3) $$\Gamma_{s,g}=\left\{s\cdots x_{i},x_{i+1}\cdots g\right\}$$

Any two adjacent waypoints $x_{i},x_{i+1}$ are assumed to be connected by a straight line segment. The path planner solves the optimal sequence of waypoints to minimize energy consumption. This formation allows waypoints to be moved on the edges rather than to be fixed at the center or vertex of the mesh. It solves the problem of discrete state transitions and guarantees path optimization. Path formation is shown in Figure 1, where the solid point represents the waypoints, and the dotted line represents the path $\Gamma_{s,g}$.

#### 2.3.2. Path Evaluation

The fitness value of each path is evaluated by measuring energy consumption. This work is primarily concerned with finding the optimum trajectory adjustments to exploit the currents fields and ensure that the vehicle does not collide with the obstacles.

1. Energy consumption

The energy consumption *E* along a given path is the sum of $e_{i}$ (energy consumption of *i*th path segment) required to cover each of the constituent path segments.

(4) $$E=\sum_{1}^{\lambda }e_{i}=\sum_{1}^{\lambda }P_{vehicle}\cdot t_{i}=\sum_{1}^{\lambda }c_{d}{\left|{\vec{v}}_{c}\right|}^{3}\cdot \frac{\left|x_{i}-x_{i-1}\right|}{\left|{\vec{v}}_{g}\right|}$$

where $P_{vehicle}$ is the vehicle’s thrust power which is proportional to the cube of the vehicle’s thrust speed. $c_{d}$ is the drag coefficient based on the vehicle’s design. ${\vec{v}}_{c}$ and ${\vec{v}}_{g}$ represent the vehicle’s thrust velocity and the velocity relative to the seabed, respectively. The vehicle’s thrust power and thrust speed are assumed to be constant (when $P_{vehicle}$ is constant, the time-optimal path is equal to the energy-optimal path). The velocity relative to the seabed is obtained by vector synthesis as the following formula.

(5) $${\vec{v}}_{g}\;=\;{\vec{v}}_{c}+\vec{c}$$

where $\vec{c}$ represents the ocean current vector, which is known either by measurement or forecasting, as described in Section 2.1.

**Figure 2:**
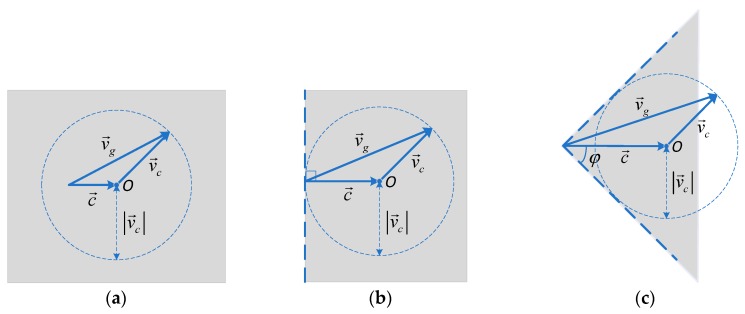
Reachable region. The stronger the current is, the more the reachable region (gray region) by the vehicle reduces. (**a**) $\left|\vec{c}\right|$ = 0.3 m/s. (**b**) $\left|\vec{c}\right|$ = 0.5 m/s. (**c**) $\left|\vec{c}\right|$ = 0.7 m/s. The vehicle’s thrust speed is $\left|{\vec{v}}_{c}\right|$ = 0.5 m/s.

It is worth noting that the operation of AUV will be significantly interfered by ocean currents. This interference limits the motion direction of AUV to a reachable region. The stronger the ocean current is, the smaller the range of the reachable region is. As shown in Figure 2a, the radius of circular *O* is $\left|{\vec{v}}_{c}\right|$. According to the velocity composition law, the range of direction of ${\vec{v}}_{g}$ is [0,2π] with $\left|\vec{c}\right|<\left|{\vec{v}}_{c}\right|$, that is, the reachable region by the vehicle is the whole plane. Similarly, the reachable region by the vehicle is a half plane with $\left|\vec{c}\right|=\left|{\vec{v}}_{c}\right|$, which is delimited by a line normal to $\vec{c}$ (see Figure 2b). And if $\left|\vec{c}\right|>\left|{\vec{v}}_{c}\right|$, the reachable region is an angular sector Φ=[−φ,φ] (see Figure 2c) with

(6) $$\varphi =\arccos\left(\frac{\sqrt{{\left|\vec{c}\right|}^{2}-{\left|{\vec{v}}_{c}\right|}^{2}}}{\left|\vec{c}\right|}\right).$$

**Figure 3:**
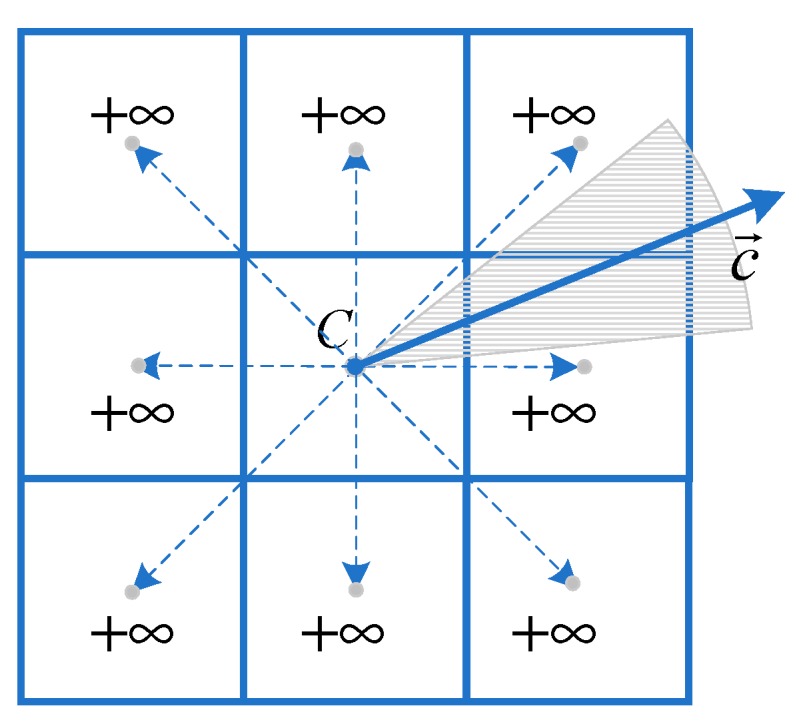
The intersection of the reachable region and allowed motion directions based on traditional path planning algorithm is empty. Some of the physically feasible moves exist in the continuous domain (gray sector). Nevertheless, all neighbors of C are tagged as unreachable in the discrete domain.

Further, if the intersection of the reachable region and allowed motion directions based on traditional path planning algorithm is empty, the path planner will return no path. Nevertheless, the physically feasible path exists, as shown in Figure 3. This is explained in detail in Reference [8]. The proposed BIO scheme solves this problem with higher computational efficiency than SWE scheme.

2. Collision constraint

Any proposed path that passes through the region of uncertainty surrounding any obstacle is discarded as unsuitable. The following expression imposes the collision value:

(7) $$C=\left\{ \begin{array}{ll} 1, & O\left(\mu_{o}^{k},\varpi_{o}^{k}\right)\\ 0, & otherwise \end{array}\right.$$

where $O\left(\mu_{o}^{k},\varpi_{o}^{k}\right)$ is the circular region of the position uncertainty at time *k* around the obstacle with radius $\varpi_{o}^{k}$ and center at $\mu_{o}^{k}$.

The collision constraint is a high priority objective that should be checked first, and its value must be zero. The evaluation function *F* is defined as

(8) $$F=\left\{ \begin{array}{ll} \inf, & C=1\\ E, & C=0 \end{array}\right.$$

## 3. Bilevel Programming Mechanism

Bilevel optimization is a branch of optimization that involves a nested optimization problem within the constraints of an upper level optimization problem [32]. The key difference between bilevel programming problems and other optimization problems are their nested structures.

In solving the problem of path planning for AUV based on grid environment, two steps are performed. The first step is to find a set of connected meshes to constitute the channel, $x_{channel}$ from the initial position to the destination with discrete state transitions for the upper level, while avoiding the mesh that contains the obstacle in the upper level. It is worth noting that the upper level problem is a combinatorial optimization. The second step is to find the exact energy-optimal path, $x_{path}$ that is constrained inside the given channel generated by the upper level in the lower level. The ($x_{channel}$, $x_{path}^{*}$) pair, where $x_{path}^{*}$ is an energy-optimal path response to $x_{channel}$, represents a feasible solution to the path planning problem. Note that most grid-based path planners only execute the first step and, thus, obtain incomplete and suboptimal paths. An example of a path generated by the bilevel optimization is shown in Figure 1. The shaded meshes represent a channel, and the dotted line is the optimal path inside this channel.

**Figure 4:**
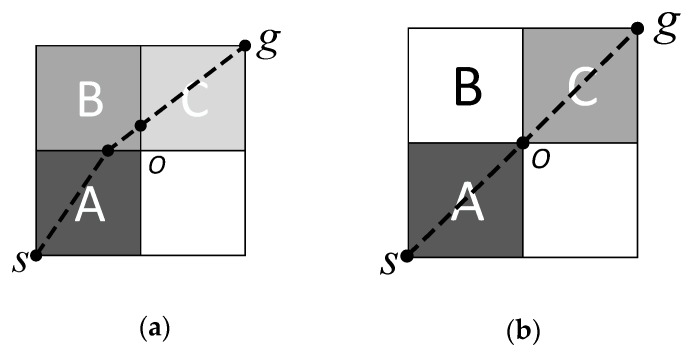
The ABC and AC are both channels from *s* to *g*. (**a**) 4-connectivity model; (**b**) 8-connectivity model.

In our study, the 4-connectivity extending model is used in the upper level, which guarantees search space integrity; it excludes the redundant channel to improve the search efficiency. As shown in Figure 4, both *ABC* and *AC* represent the channel from the initial position *s* to the destination *g*. In the 4-connectivity extending model, only channel *ABC* can be generated. By contrast, *ABC* and *AC* can be both generated in an 8-connectivity extending model, although channel *AC* is redundant. Channel *AC* contains the unique path {s,O,g} because mesh *A* and mesh *C* are connected only through vertex *O*, which does not satisfy the definition of the channel. The path {s,O,g} can be found in the channel *ABC* when the two waypoints at the borders of mesh *B* coincide with point *O*. Therefore, the 4-connectivity extending model is suitable for generating the channel.

We solve the above problem by using a nested bilevel optimization algorithm, where the upper level problem is solved by the ACA and the lower level problem is solved by the QPSO. ACA is suitable for solving the combinatorial optimization problems while QPSO is suitable for solving the multivariable optimization problems. The process for the algorithm is described as follows:

Step 1: Initialization. Choose the appropriate weight coefficient for α (the coefficient that characterizing the importance of pheromone), β (the coefficient that characterizing the importance of the heuristic information), and ρ (pheromone evaporation coefficient). The data, including the number of ants *m* (equivalent to the number of candidate channels), the current number of iterations $N_{c}$, the maximum number of iterations $N_{\max}$, the heuristic information matrix *H*, which is equivalent to the reciprocal of distance between the current position and the destination, and initial pheromone matrix *T*, are initialized. Then, the ocean field information is inputted.

In the problem of path planning, few meshes receive pheromones in some cases because of the large scale of the problem (a 50 × 50 environment model has 2500 meshes). This condition leads to a serious problem wherein optimization falls into a local solution. So, the values of α and β are adjusted as

(9) $$\alpha =\left\{ \begin{array}{ll} \frac{4\cdot N_{c}}{N_{m}}, & if\;0\leq N_{c}<N_{m}\\ 4, & if\;N_{m}\leq N_{c}\leq N_{\max} \end{array}\right.$$

(10) $$\beta =\left\{ \begin{array}{ll} \frac{20\cdot N_{m}-10\cdot N_{c}}{N_{m}}, & if\;0\leq N_{c}<N_{m}\\ 10, & if\;N_{m}\leq N_{c}\leq N_{\max} \end{array}\right.$$

where $N_{m}$ is the critical number of iterations. If $0\leq N_{c}<N_{m}$, the dominant factor in ant routing is regarded as heuristic information because of the relatively small number of pheromones on each mesh. At this stage, the ant finds the destination more easily, and more meshes can get pheromones. Then, the dominant factor in ant routing is changed into pheromone factor when $N_{m}\leq N_{c}\leq N_{\max}$. This adjustment is effective for path planning and preventing falling into a local solution.

Step 2: Constructing channels at the upper level. When an ant constructs a feasible channel, it must crawl through a set of connected meshes from an initial position to the destination. A given probability selection formula is then applied to determine the selection probability of each available meshes, where meshes with transfer values are selected based on certain rules. The ant *k* in mesh *i* can calculate the probability of visiting mesh *j* according to Equation (11). The criterion of exploration depends on two terms, one relating to heuristic information and the other relating to the quantity of pheromones deposited by all the ants.

(11) $$p_{ij}^{k}=\left\{ \begin{array}{ll} \frac{\tau_{ij}{}^{\alpha }\cdot h_{j}{}^{\beta }}{\sum_{j\in allowed\left(i\right)}\tau_{ij}{}^{\alpha }\cdot h_{j}{}^{\beta }} & j\in allowed\left(i\right)\;and\;j\notin tabu\left(i\right)\\ 0, & else \end{array}\right.$$

where $\tau_{ij}$ is the concentration of pheromones on channel (*i*, *j*) which consists of meshes *i*, *j,* and $h_{j}$ that represents heuristic information of mesh *j*. The *allowed*(*i*) represents a set of meshes that can be explored and do not contain the obstacle. The *tabu* list represents the set of meshes that the ant has already passed through. The records in *tabu* list change as the ants select different channels.

Step 3: Optimization at the lower level. For each of the generated channels at the upper level, preform the lower level optimization to determine the exact energy-optimal path by QPSO, as described in Algorithm 2. The resulting channels of the upper level programming as the constraint of the lower level optimization. The individuals are evaluated based on the energy consumption function Equation (4). Finally, the procedure returns the best value, $E_{k}^{*}$ of the lower level optimization.

Step 4: Releasing pheromones. According to fitness, pheromone is released according to certain proportions. The higher the fitness, the more pheromones are released. The pheromone updating mechanism is represented by the following equation:

(12) $$\tau_{ij}\left(N_{c}\right)=\left(1-\rho \right)\cdot \tau_{ij}\left(N_{c}-1\right)+\sum_{k=1}^{m}\Delta \tau_{ij}^{k}$$

where $\Delta \tau_{ij}^{k}$ represents the released pheromone of the ant *k* on the channel (*i, j*). The expression is as follows:

(13) $$\Delta \tau_{ij}^{k}=\left\{ \begin{array}{ll} \frac{Q}{E_{k}^{*}}, & \left(i,j\right)\in x_{channel}\\ 0, & \left(i,j\right)\notin x_{channel} \end{array}\right.$$

where *Q* represents the pheromone increasing coefficient, which is a constant. Equation (13) is the pheromone update calculation method based on the ant-cycle model. This method updates pheromones for the global channel, making it highly efficient and effective.

Step 5: Termination check. A termination check is performed before the next generation (Step 2) if the termination check is false.

The Algorithm 1 shows the simplified upper level algorithm.
**Algorithm 1.** Upper level algorithmInitialization: Input: initial position, destination and the current information of each mesh Input: choose appropriate value for α, β, ρ, Q, $N_{\max}$ and the number of ants m. Setting initial the heuristic information matrix H and pheromone matrix T Main loop: **while** the terminate condition is not met do **for** each ant k **do** **while** ant k finds a channel from initial position to destination **do** choose next mesh with probability selection Equation (11). update the *tabu* list **end while** optimization at the lower level and computing the energy consumption $E_{k}^{*}$ of the tour constructed by the kth ant   **end for** update the pheromone matrix T **end while**

Algorithm 2 provides an overview of the iterative QPSO algorithm for the lower level optimization. Every particle in the swarm represents a potential path, and the parameters of each particle correspond to the coordinates of the waypoints generating the path. As the QPSO algorithm iterates, each particle is attracted towards its respective local attractor according to the outcome of the particle’s individual search, as well as the particle swarm’s search results.
**Algorithm 2.** Lower level algorithm.Initialization: Choose appropriate parameters for the population size, *n*, (equivalent to the number of candidate paths), the current number of iterations *X_c_* and the maximum number of iterations *X*_max_. Set *X_c_* = 1. Generate an initial group of particles with random states representing the candidate paths. Initialize the current state *P_i_*(0) and the *pbest* state *𝒫_i_*(0) = *P_i_*(0). Main loop: **while** the terminate condition is not met **do** Compute the mean best state
(14)$$mbest\left(X_{c}\right)=\sum_{i=1}^{n}\frac{P_{i}\left(X_{c}\right)}{n}$$
**for** each particle *i*
**do** Evaluate the cost function *F*(*P_i_*(*X_c_*)) as defined in Equation (8); Update the *pbest* state *𝒫* and the *gbest* state *G*;
(15)$$P_{i}\left(X_{c}\right)=\left\{ \begin{array}{ll} P_{i}\left(X_{c}-1\right), & \;\mathrm{if}\;F\left(P_{i}\left(X_{c}\right)\right)\geq F\left(P_{i}\left(X_{c}-1\right)\right)\\ P_{i}\left(X_{c}\right), & \;\mathrm{if}\;F\left(P_{i}\left(X_{c}\right)\right)<F\left(P_{i}\left(X_{c}-1\right)\right) \end{array}\right.,$$
(16)$$G\left(X_{c}\right)=\underset{1\leq i\leq n}{\arg\min}F\left(P_{i}\left(X_{c}\right)\right)$$
**for** each dimension of particle *j*
**do**
(17)$$\tau =\frac{\left(1.0-0.5\right)\cdot \left(X_{\max}-X_{c}\right)}{X_{c}}+0.5$$
(18)$$\upsilon_{i,j}\left(X_{c}\right)=\varphi_{i,j}\cdot P_{i,j}\left(X_{c}\right)+\left(1-\varphi_{i,j}\right)\cdot G_{j}\left(X_{c}\right)\varphi_{i,j}∼U\left(0,1\right)$$
(19)$$\psi_{i,j}\left(X_{c}\right)=\tau \cdot \left|mbest_{j}\left(X_{c}\right)-p_{i,j}\left(X_{c}\right)\right|$$
(20)$$p_{i,j}\left(X_{c}+1\right)=\left\{ \begin{array}{l} \upsilon_{i,j}\left(X_{c}\right)+\psi_{i,j}\left(X_{c}\right)\cdot \ln\frac{1}{\varphi_{i,j}},\;\mathrm{if}\;\varphi_{i,j}\geq 0.5\\ \upsilon_{i,j}\left(X_{c}\right)-\psi_{i,j}\left(X_{c}\right)\cdot \ln\frac{1}{\varphi_{i,j}},\;\mathrm{if}\;\varphi_{i,j}<0.5 \end{array}\right.$$
**end for** **end for** Set $X_{c}=X_{c}+1$; **end while** Return *G* as the optimal fitness value and its correlated path as the optimal solution to the upper level programming.

In algorithm 2, the state of the *i*th particle is represented as follows

(21) $$P_{i}=\left[p_{i1},\cdots ,p_{ij},\cdots ,p_{ik}\right]$$

where $p_{ij}$ represents the position of the waypoint at the boundary of *j*th mesh and (*j* + 1)th mesh in *i*th channel. The dimension *k* of every particle is determined by the number of meshes *M* contained in the channel. The relationship between them is

(22) k=M−1

The Contraction-Expansion coefficient is represented as τ, which is the only parameter in QPSO algorithm. It can be tuned to control the convergence speed of the algorithms. $\varphi_{i,j}$ is random number distributed uniformly on [0, 1]. $\psi_{i,j}$ is called as the potential well length, which represents the scope of searching of particles.

## 4. Simulation Results

The simulation results obtained for the energy-optimal path planning problem through different scenarios are shown. To evaluate the performance of the proposed bilevel optimization scheme, we have selected the SWE algorithm as the benchmark, which is a deterministic algorithm based on continuous optimization technique. The algorithms are implemented by using MATLAB 2014a on an Intel Core i5 processor with a speed of 3.2 GHz × 4 and 8 GB of RAM.

### 4.1. Simulation Setup

As mentioned in Section 2.1, the current field is computed from a random distribution of 20 vortices represented by a 50 × 50 grid. The range of the random values $size_{i}$ is set as [−0.3, 0.3]. The mean of ocean current is between 0.2 m/s and 0.4 m/s, and the maximum ocean current is between 0.7 m/s and 1.0 m/s in our simulation scenarios. The distance between the nearest neighbor grid points corresponds to 1 km. The initial and final destination points are located at the center of mesh (1, 1) and (50, 50). We use the NACA series 58 body as the model, where $c_{d}=0.0064$ and $\left|{\vec{v}}_{c}\right|=0.5\;m/s$.

The experimentally optimized settings of the BIO scheme are as follows:

(1) ACA (the upper level): the ant number is 20, and the maximum number of iterations is 100. The pheromone evaporation coefficient ρ and pheromone increasing coefficient *Q* is equal to 0.3 and 10, respectively. The values of α and β is determined by Equations (9) and (10), respectively. The critical number of iterations $N_{m}$ is 30.

(2) QPSO (the lower level): the population size is 50, and the maximum number of iterations is 500.

The additional stop criterion of both levels is satisfied when the weighted average change in the fitness function value over a set number of iterations is less than the function tolerance (1 × 10^−5^), as follows:

(23) $$W=\sum_{i=1}^{20}\left(\frac{E_{l-i}-E_{l-i-1}}{E_{l-i}}\right){\left(0.5\right)}^{i-1}$$

where *l* is the number of the current iteration and *E* is the relevant fitness value.

To better compare, the modified SWE algorithm is used in following simulation experiments, which the projected gradient method (similar to the lower level optimization) in SWE algorithm is replaced by QPSO with the same settings as the BIO.

### 4.2. Simulation Experiments with Different Scenarios

**Figure 5:**
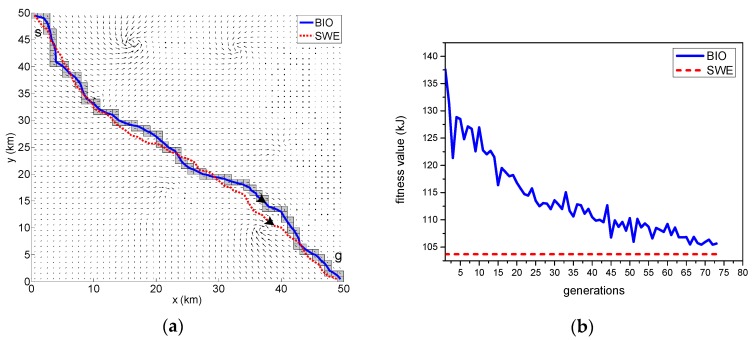
Comparison of results produced by the bilevel optimization (BIO) and sliding wavefront expansion (SWE) schemes in the none obstacle environment: (**a**) the optimal path generated by BIO and SWE schemes. (**b**) convergence curve of the upper level of BIO scheme.

**Figure 6:**
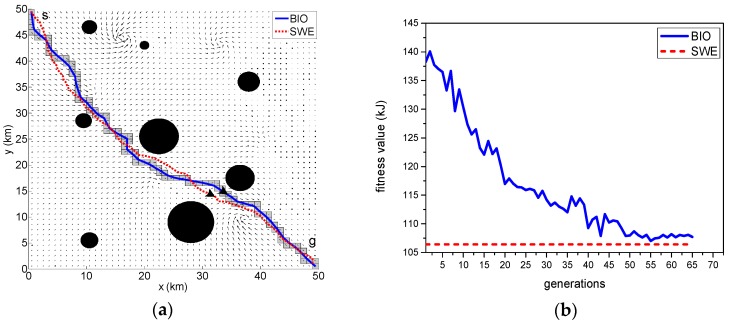
Comparison of results produced by the BIO and SWE schemes in the environment with fixed uncertainty obstacles: (**a**) the optimal path generated by BIO and SWE schemes. (**b**) convergence curve of the upper level of BIO scheme.

**Figure 7:**
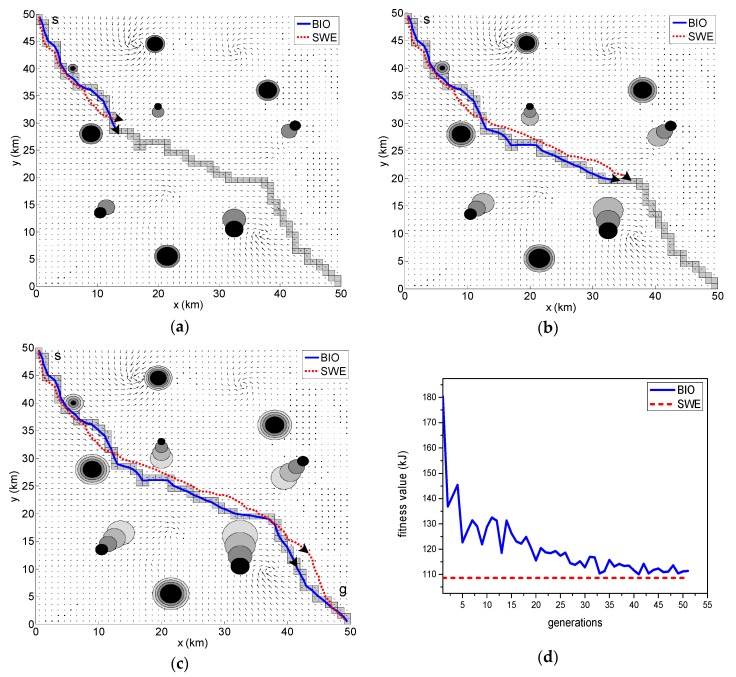
Comparison of results produced by the BIO and SWE schemes in the environment with varying uncertainty obstacles: (**a**–**c**) the optimal path generated by BIO and SWE schemes in 3 time steps. (**d**) convergence curve of the upper level of BIO scheme.

The results of the path optimization with the same currents field are displayed in Figure 5a, Figure 6a and Figure 7a–c. The optimal paths are respectively generated by the BIO scheme and SWE scheme. The solid line located in the channel represents the result of path generated by the BIO, and the dotted line represents the optimal path obtained by the SWE.

Figure 5a shows the optimal path in a scenario with no obstacles. Figure 6b displays the result of the optimal path in a scenario containing randomly distributed static obstacles with fixed uncertainty. The position uncertainty of each obstacle is represented as a black circle around the obstacle with radius $2\sigma_{o}$ and indicates that the obstacle is located within this area at a confidence of 95.4%. The safe trajectory is achieved when the vehicle maneuver does not have any intersection with the proposed obstacle boundary. Figure 7a–c shows the simulation results of the scenario with quasi-static and moving obstacles. The uncertainty over both the quasi-static and moving obstacles are linearly propagated relative to the updating time. This leads to the radius growth of the static obstacles and simultaneous position and radius changes in the moving obstacles, which is expressed as a proportional increment in the collision boundary encircling the obstacles. The varying uncertainty of the obstacles can be clearly seen in the subsequent Time Step 1–3 of Figure 7.

Evidently, the utilized BIO and SWE path planning methods are capable of generating a collision-free path against the distribution of obstacles and taking advantage of ocean current to minimize the vehicle’s energy consumption.

Table 1 lists the best fitness value and computation time of the two methods in finding the optimal solution considering the augmented objective function Equation (4). By comparing the simulation results, we have found that the computation time of BIO scheme is significantly less than that of the SWE scheme, and there is a bit of difference (less than 2%) in best fitness value between two schemes.

The convergence curves of the upper level of the BIO scheme is shown in Figure 5b, Figure 6b, and Figure 7d, in which the broken lines represent the result of SWE scheme. The algorithm does not preserve the elite member in the upper level, so continuous improvement is not observed. Instead, some humps are contained. These convergence curves show that the fitness values are close to the optimal value given by the SWE algorithm during 30–45 iterations and the stopping criterion is satisfied during 50–75 iterations. It demonstrates that the proposed BIO algorithm provides good performance in terms of convergence. In addition, it can be seen that the iteration count decreases as the complexity of the scenario increases. This is because that the increase in complexity reduces the search space.

In addition, the A*-QPSO scheme (A* as the upper level optimization and QPSO as the lower level optimization) was tried for the outstanding performance of A* in the field of path planning. The five sets of results about SWE, A*-QPSO and BIO are listed in Table 2 in the none obstacle environment. Since the best fitness values of SWE and A*-QPSO are approximately equal (slight differences are caused by the uncertainty of QPSO), only computing time is shown.

As can be seen from Table 1, the performance of A*-QPSO is better than SWE scheme but worse than BIO scheme. The reasons are as follows:

According to A* algorithm theory, the performance of A* algorithm is largely determined by its heuristic function. The closer the heuristic function is to the actual value, the better the computational efficiency of A* algorithm is. In the field of path planning for a land robot or computer games, the heuristic usually is defined as the distance between current position and goal position. However, in the problem of energy-optimal path planning for AUV, the heuristic function will be adjusted according to the ocean current fields. The reasonable heuristic function is $h\left(x_{i}\right)=c_{d}\cdot {\left|{\vec{v}}_{c}\right|}^{3}\cdot \frac{\mathrm{distance}\;(\mathrm{from}\;\mathrm{current}\;\mathrm{position}\;\mathrm{to}\;\mathrm{goal}\;\mathrm{position})}{\left|{\vec{v}}_{c}\right|+\left|{\vec{c}}_{\max}\right|}$, where ${\vec{v}}_{c}$ and ${\vec{c}}_{\max}$ respectively represent the thrust velocity of vehicle and the largest ocean current. The introduction of the largest ocean current (which is to guarantee $h\left(x_{i}\right)<g\left(x_{i}\right)$, where $h\left(x_{i}\right)$ and $g\left(x_{i}\right)$ represent the heuristic value and actual cost value from $x_{i}$ to goal point, respectively.) reduces the weight of heuristic factors. The guiding effect of heuristic factors is weakened. Therefore, compared with A* algorithm without considering the current field, the advantages of A* are not well represented.

Based on the above reasons, this paper does not use A* approach as the path planning algorithm, and the further simulation for A*-QPSO is not implemented.

### 4.3. Performance Assessment

To evaluate the performance of the proposed algorithm, we have performed the simulations on the basis of randomly generated ocean currents (*N* = 100) with various obstacles. The settings for each simulation are the same as those described in Section 4. The performance of SWE and BIO are compared according to the following factors: the best fitness value and computation time. The best fitness value and the computation time respectively reflect the searching ability and searching efficiency.

Table 3 shows that the BIO scheme generates paths at significantly shorter computation time, which the computation time of BIO scheme is about 1/3 of SWE scheme, and a slightly worse fitness value (less than 5%, which is acceptable because the errors also exist in vehicle navigation and ocean current measurement) than the SWE scheme. The differences in the mean of best fitness value are respectively 3.8%, 2.9%, and 4.3% under the three scenarios. It is worth noting that although the SWE scheme is used as a benchmark algorithm, the best fitness value of BIO scheme is possibly smaller than the value of SWE. This is because that the QPSO as a random searching algorithm has uncertainty. The last column in Table 3 provides information about QPSO calls to count. It shows that SWE took more QPSO evaluations than the BIO scheme and thus the SWE scheme has a long computation time. Note that the QPSO calls count is not equal to the product of the number of iterations and the quantity of ants because some ants may be a failure to find the channel to the destination especially in the initial stage of searching.

## 5. Conclusions

The BIO scheme is presented to solve the problem of path planning for AUV. The scheme works by splitting the path planning task into a selection channel and optimization path in the selected channel. The ACA and QPSO are used as the upper level and lower level algorithms, respectively. As indicated by the results obtained at different scenarios, the BIO scheme is capable of finding a collision-free path, while taking advantage of the ocean current to reduce energy consumption. We have compared the results obtained using the BIO scheme with that obtained by the SWE scheme and have found that the BIO scheme considerably improves computation efficiency with an acceptable accuracy.

## Figures and Tables

**Table 1:** Performance comparison of bilevel optimization (BIO) and sliding wavefront expansion (SWE) with different scenarios.

Obstacles Form	Algorithm	Best Fitness Value (kJ)	Computation Time (s)
None obstacle	SWE	103.7	1275
	BIO	105.4	459
Fixed uncertainty	SWE	106.4	1257
	BIO	107.4	403
Varying uncertainty	SWE	108.6	1191
	BIO	110.0	320

**Table 2:** Comparison of computing time of SWE, A*- quantum-behaved particle swarm optimization (QPSO) and BIO.

Algorithm	Computing Time (s)				
SWE	1270.7	1423.7	1321.8	1621.8	1362.0
A*-QPSO	1150.0	1132.0	1130.7	1224.0	1129.8
BIO	411.4	409.6	409.4	406.6	401.7

**Table 3:** Performance comparison of BIO and SWE based on 100 runs.

Obstacles Form	Algorithm	Best Fitness Value (kJ) (mean)	Computation Time (s) (mean)	QPSO Calls Count (mean)
None	SWE	111.1	1372	15,325.9
	BIO	115.3	481	994.8
Fixed uncertainty	SWE	113.7	1347	14,388.4
	BIO	117.0	384	889.4
Varying uncertainty	SWE	112.9	1354	14,309.5
	BIO	117.7	365	847.7

## Abbreviations

BIO	bilevel optimization
GA	Genetic algorithm
SWE	The sliding wavefront expansion algorithm
ROMS	the Regional Ocean Model System
QPSO	The quantum-behaved particle swarm optimization
AUV	Autonomous underwater vehicle
ACA	The ant colony algorithm
FM	The fast marching algorithm
FM*	Heuristically guided FM
